# The Impact of Septoplasty on Eustachian Tube Function

**DOI:** 10.1055/s-0045-1812059

**Published:** 2026-04-24

**Authors:** Mohammed Radef Dawood

**Affiliations:** 1 Department of Otorhinolaryngology, College of Medicine, Mustansiriyah University, Baghdad, Iraq

**Keywords:** septoplasty, Eustachian tube function, tympanometry

## Abstract

**Introduction:**

Septoplasty is standard surgical procedure performed for correction of deviated nasal septum; it affects middle ear ventilation through altering Eustachian tube function.

**Objective:**

To determine the effect of septoplasty on Eustachian tube function regarding deviation side.

**Methods:**

A randomized, prospective controlled trial, in which 40 adult patients who underwent septoplasty (80 ears) were divided into: group A: 40 ears in which Eustachian tube function assessment was done on the affected side (deviated nasal septum), and 40 ears in which the function of Eustachian tube assessment was done on the contralateral side “group B”. The nasal obstruction symptom evaluation (NOSE) scale was used to analyze surgical satisfaction, ventilation of middle ear was assessed via Eustachian tube functions by the 7-Item Eustachian Tube Dysfunction Questionnaire (ETDQ-7) test scale, insufflation tests (Valsalva and Toynbee), and tympanometry. These parameters were analyzed and compared, before and after septoplasty.

**Results:**

Among the 40 patients, the mean NOSE score was 13.68 ± 2.69 preoperatively and decreased to 5.76 ± 4.48 postoperatively; ETFQ-7 scores decreased from 12.48 ± 4.78 preoperatively to 7.56 ± 3.4 postoperatively; there were 22 functional Eustachian tubes (55%) preoperatively, which increased to 37 (92.5%) postoperatively, while dysfunctional Eustachian tubes decreased from 18 (45%) preoperatively to 3 (7.5%) postoperatively. Type-A curve tympanogram was (52.5%) preoperatively, which increased to (96.25%) postoperatively, while type-C tympanogram was (47.5%) preoperatively and decreased to (3.75%) postoperatively. Basal middle ear pressure was -33.56 daPA for group A and -29.24 daPA for group B preoperatively, and it changed to -18.96 daPA and −12.18 daPA postoperatively, respectively.

**Conclusion:**

Septoplasty had a beneficial impact on the Eustachian tube function “ventilation of middle ear”.

## Introduction

The Eustachian tube (ET) connects the cavity of the middle ear to the nasopharynx and provides middle ear ventilation, as the physiological pressure depends on the air transition through the ET and gas diffusion between the mucosa of the middle ear and the systemic circulation. Thus, functional ET is crucial for balancing the middle ear pressure. Eustachian tube dysfunction can occur due to obstruction of its orifice in those with deviated nasal septum (DNS). This causes increased secretion from the nasal glands that accumulate and block the tube, thus leading to reduced middle ear ventilation, which causes the pressure in the middle ear to be negative. 1

Although there is no gold standard method used to evaluate ET function, many different methods are used to do so, such as sonotubometry, which is based on the measurement of sound waves that pass through the ET; however, this method can be challenging. Another modality is the manometry test, which has its limitations. Since the evaluation of ET functions was insufficient with the tympanic membrane intact, insufflation tests (Valsalva or Toynbee maneuver) and tympanometry are not difficult to perform and are widely used to evaluate ET function clinically, as well as in academic research. 2 Questionnaires, such as the 7-Item Eustachian tube dysfunction questionnaire-7 (ETDQ-7), are also used for screening. 3

The question that arises is if treatment for DNS can improve patients' hearing by improving ET function and middle ear ventilation. Few studies are available in literature regarding this topic, and they have obtained varied results; yet it is well known that septoplasty is the most common surgery performed in the otorhinolaryngology practice for DNS. 4

Therefore, the aim of the current study was to analyze the effect of DNS correction septoplasty on middle ear ET function on the side of deviation.

## Methods

A randomized prospective controlled trial was conducted in the Otolaryngology Department of Mustansiriyah University. Patientss' written consent as well as Institutional Ethics Committee approval were obtained.

Forty adults underwent septoplasty; therefore, 80 ears were evenly and randomly divided into 2 groups: group “A,” in which the ET functions of 40 ears were assessed on the affected side (side of DNS), and group “B,” which contained 40 ears in which the Eustachian tube functions were assessed on the contralateral side.

All patients with perforated tympanic membrane, active middle ear disease, external ear disease, nasal polyps, allergic rhinitis, chronic sinusitis, or active upper respiratory tract infection as well as those who previously underwent septoplasty, endoscopic sinus surgery, and those who were unfit for general anesthesia, or did not attend regular follow-up were excluded from the current study. Other excluded groups were children and patients with DNS that causes bilateral nasal obstruction (such as s-shaped septal deviation or patients with compensatory hypertrophy of the inferior turbinate).

Septoplasty was performed due to unilateral DNS, under general anesthesia, with the same surgical technique and by same surgical team, with no additional surgical procedures.

Surgical satisfaction was evaluated by the nasal obstruction symptom evaluation (NOSE) scale, which is a 0-to-4-point scale, as follows: 0: not a problem; 1: very mild problem; 2: moderate problem; 3: fairly bad problem; and 4: severe problem.

Middle ear ET function was assessed with the ETFQ-7 test scale, tympanometry, and insufflation tests (Valsalva and Toynbee).

The data was obtained through interview. The ETDQ-7 questionnaire was applied preoperatively 12 weeks after surgery. The investigators who did the interviews were blind to the study. The time needed to complete the questionnaire was limited to between 10 and 12 min, and the interviewer respected the Arabic native language.

**Fig. 1:**
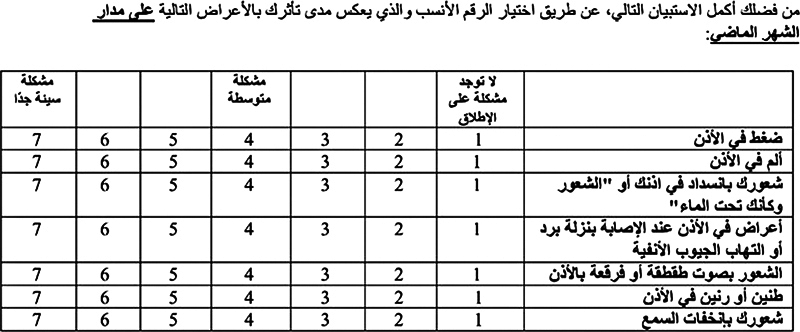
Arabic version of the 7-Item Eustachian Tube Dysfunction Questionnaire (ETDQ-7).

First, the Arabic version of the ETDQ-7 questionnaire was validated and then translated back to English language. This questionnaire consists of 7 items with a scale ranging from 1 to 7, thus allowing quantitative subjective measurement. The 7 items are: (1) ear pressure; (2) ear pain; (3) feeling the ears are clogged or under water; (4) ear-related symptoms in case of cold or sinusitis; (5) cracking or popping sounds; (6) ear ringing; and (7) muffled hearing. A score lower than 14.5 was a normal result. Fig. 1 shows the Arabic version of the ETDQ-7 questionnaire.

## Tympanometry

A tympanometry evaluation of the ET was performed with the AZ 26 (Interacoustics A/S) middle ear analyzer. Initially, the first basal tympanogram was obtained on the operative day and then, the tympanometry peak pressures (TPPs) were analyzed after performing the Valsalva and Toynbee maneuvers to assess the ET function; the Valsalva maneuver was usually done by closing the mouth and nose, in order to forceful attempt opening the ET, while the Toynbee maneuver was done via swallowing motion with the nose closed, which leads to pressure changes in the middle ear. The TPP was functional when a TPP change ≥ 10 Deca pascals (daPa) was observed; if the alteration was < 10 daPa, the ET function was poor.

The assessment was repeated at 4 and 8 weeks, and the final assessment at 12 weeks postoperatively, and these results were compared and analyzed.

## Statistical Analysis

Statistical analyses were performed with the IBM SPSS Statistics for Windows (IBM Corp.) software, version 25.0. Descriptive statistics considered mean and standard deviation for continuous measures, counts and percentages for categorical variables, and the Mann-Whitney test was used to compare the means of two groups. A Spearman's correlation was used to correlate continuous variables, a paired t-test was applied to compare the pre and postoperative results, with standard deviation and confidence determined as 95%, and a *p* -value < 0.05 considered statistically significant.

## Results

Forty patients aged > 18 years were included in the current study; the mean age was 28.35 ± 16.86 years, with the most common age group being 18 to 30 years (60%). There were 23 males (57.5%) and 17 females (42.5%), which was found not to be statically significant ( *p* -value > 0.05), as shown in Table 1 .

**Table 1:** Age and sex distribution

Age group (years)	Male	Female	Total	Percentage
18–29	14	10	24	60%
30–39	5	4	9	22.5%
40–49	3	2	5	12.5%
50–59	1	1	2	5%
Total	23	17	40	100%

The 40 adult patients underwent isolated septoplasty. The mean NOSE score was 13.68 ± 2.69 before surgery and 5.76 ± 4.48 after surgery. The NOSE score at 12 weeks postoperatively was statistically significant as the *p* -value was < 0.011.

Among the 40 patients studied (80 ears), there were 22 (55%) functional ETs preoperatively, which increased to 37 (92.5%) postoperatively. Meanwhile, dysfunctional ETs were seen in 18 (45%) patients preoperatively, which decreased to 3 (7.5%) at the 12-week postsurgery assessment. These improvements were statistically significant ( *p* < 0.05). In addition to that, the mean NOSE score was 13.68 ± 2.69 before septoplasty and 5.76 ± 4.48 postoperatively, which was statistically significant ( *p* = 0.01). Table 2 shows these findings per group.

**Table 2:** NOSE scores and the number of Eustachian tube evaluations before and after surgery between the study groups

	Before septoplasty	After septoplasty
Mean NOSE score	13.68 ± 2.69	5.76 ± 4.48
Statistically significant results via paired t-test, with *p* -value < 0.011		
Group A	Functional ET 8 ears (20%)	Functional ET 38 ears (95%)
Statistically significant via paired t-test, with *p* -value < 0.01		
Group B	Dysfunctional ET 8 ears (20%)	Functional ET 36 ears (90%)
Statistically significant via paired t-test, with *p* -value < 0.01		

**Abbreviations** : ET, Eustachian tube; NOSE, nasal obstruction symptom evaluation.

There was marked improvement at 8 (37.5%) and at 12 (15%) weeks postoperatively, among the 34 patients (85%) who reported ear fullness at the first appointment; however, no marked improvement was observed for earache and tinnitus as shown in Table 3 .

**Table 3:** Main otological symptoms' assessment via ETFQ-7 scores

Ear symptoms	Preoperative n = 40 (%)	Assessment after 4 weeks n = 40 (%)	Assessment after 8 weeks n = 40 (%)	Assessment after 12 weeks n = 40 (%)
Ear fullness	34 (85%)	19 (47.5%)	15 (37.5%)	6 (15%)
Earache	7 (18%)	(15%)6	5 (12%)	5 (12%)
Tinnitus	10 (25%)	9 (23%)	8 (20%)	8 (20%)

Abbreviation: ETDQ-7, 7-Item Eustachian Tube Dysfunction Questionnaire.

## Tympanometry Findings

In group A (affected side), a type-A tympanometry curve was detected in 5 ears (12.5%), and a type-C curve was seen in 35 ears (87.5%) preoperatively. These became 39 ears (97.5%) with type-A curve and only 1 ear (2.5%) remained as type-C curve postoperatively.

While in group B (contralateral side), a type-A tympanometry curve was found in 37 ears (92.5%) and a type-C curve was seen in 3 ears (7.5%) preoperatively. These became 38 ears (95%) with type-A curve and 2 ears (5%) with type-C curve after septoplasty.

In both groups, a type-A curve was detected in 52.5% of patients' ears, preoperatively but this number increased to 96.25% at the 12-week postoperative assessment. Meanwhile, a type-C curve, which indicates negative middle ear pressure, was seen in 47.5% of ears preoperatively but then decreased to 3.75% of patients' ears at the 12-week postoperative assessment. This data can be seen in Table 4 .

**Table 4:** Tympanometry types

Tympanometry curves types	Preoperative (number of ears = 80): n (%)	Assessment after 4 weeks (number of ears = 80): n (%)	Assessment after 8 weeks (number of ears = 80): n (%)	Assessment after 12 weeks (number of ears = 80): n (%)
A	42 (52.5%)	54 (67.5%)	63 (78.75%)	77 (96.25%)
C	38 (47.5%)	26 (32.5%)	17 (21.25%)	3 (3.75%)

The changes in tympanometry results were statistically significant in both groups, with *p* -values < 0.05. The basal middle ear pressure value was -33.56 daPA in group A and -29.24 daPA in group B. After septoplasty, the middle ear pressure in group A was −21.18 daPA and -18.96 daPA in group “B”.

In the evaluation of tympanometry peak pressures obtained after performing the Valsalva maneuver, there was a significant change observed in groups A ( *p* -value = 0.002) and B ( *p* -value = 0.023).

In addition, the postoperative change in TPPs obtained after the Toynbee maneuver was statistically significant in both groups A and B). All these data are shown in Table 5 .

**Table 5:** Evaluation of mean tympanometry peak pressures before and after septoplasty among the study groups

Study groups	Group A			Group B		
Mean results	Basal	Valsalva	Toynbee	Basal	Valsalva	Toynbee
Preoperative	-33.56 ± 13	27.48 ± 58	49.78 ± 436	-29.24 ± 71	27.55 ± 67	57.56 ± 24
4th postoperative week	-19.48 ± 25	22.13 ± 68	36.47 ± 28	-25.69 ± 34	24.64 ± 52	41.81 ± 57
8th postoperative week	-16.73 ± 63	17.56 ± 24	25.31 ± 47	-21.98 ± 16	20.76 ± 93	32.47 ± 69
12th postoperative week	-12.18 ± 52	10.78 ± 36	22.56 ± 89	-18.96 ± 27	-19.43 ± 82	14.26 ± 87
*p* -value	< 0.05	0.002	0.012	< 0.05	0.023	0.017

## Discussion

Several of the patients seen at the Ear, Nose, and Throat (ENT) Clinics suffered from middle ear ventilation problems.

Eustachian tube dysfunctions concomitant with nasal obstructive pathologies due to NSD are usually managed surgically through a nasal septal correction technique known as septoplasty. Therefore, the prevalence of NSD might reach 80%. In general, many patients have nasal congestion and ET dysfunction simultaneously, as the nasal cavity conditions influence the mucosa around the ET and can be a determining factor for ETF. That is, ET dysfunction can occur due to allergic rhinitis, chronic rhinosinusitis, or NSD. Several studies have suggested that NSD may be the reason for dysfunction of the ET. 5

The NOSE scale, which is a validated and reliable tool to evaluate nasal obstruction in adults. 6 7

In the current study, there was a slight male predominance, and the most common age group affected was the younger one, which agrees with the findings of other studies. 8 9 Gallardo et al., 10 on the other hand, noted a female predominance.

Also, in the current study, there were statistically significant findings such as: decrement in both ETFQ-7 scores and number of dysfunctional ETs, in addition to changes in tympanometry curves, mainly to type A, after septoplasty.

Nanda et al. 4 stated in their tympanometry findings that type-C tympanogram curve, which is indicative of negative middle ear pressure, was obtained in 36% of the patients' ears preoperatively and went up to 44% of patients' ears at 2 days postoperatively. However, they saw significant improvement, with only 12% of patients' ears showing a type-C curve at 12 weeks postsurgery.

Kaya et al. 5 concluded that the changes in the tympanometry results were statistically significant for both the affected (−33.56 daPA/− 21.18 daPA) and contralateral sides (29.24 daPA/− 24.96 daPA) ( *p* < 0.05), but the alteration in the side of deviation was more evident.

The findings of both studies above-mentioned agree with the results of the current study.

While a study done by Young Hoon et al. 11 reported that, among the group in which ET dysfunction was confirmed before surgery, there were 7 patients who underwent tests for ETF after surgery. In five of them, ET dysfunction resolved after surgery, and in the remaining two patients, the degree of ET dysfunction decreased. However, this result did not make a statistically significant difference.

Many studies investigated ETF after septoplasty.

In the current study, the evaluation of TPPs gained after performing the Valsalva and Toynbee maneuvers showed significant changes in both the deviated and the contralateral sides.

In the study by Kaya et al. 5 , the TPP analysis obtained after the Valsalva maneuver shows a statistically significant change on the deviated side, while no significant change was noted in the contralateral side, whereas the postoperative change in TPPs obtained after the Toynbee maneuver was not significant in either the affected or the contralateral side.

Low and Willatt 12 concluded in their study that after 40 patients underwent septoplasty, their mean TPPs lowered significantly.

Deron et al. 13 used a manometry test for ET compliance during the Valsalva maneuver to clarify the relationship between surgery for deviated nasal septum and ETF. An enhancement in the pressure of the ET opening was observed on the deviated and contralateral sides in both early and late postoperative septoplasty.

Also, a study done by Salvinelli et al. 14 subjected the analyzing functions of ET in 40 patients with the Toynbee and Valsalva maneuvers before and after septoplasty. The ETF test results improved significantly after septoplasty. A recent study done by Akyıldız et al. 15 found a higher rate of ET dysfunction in patients with DNS, and they were detected to be had an enhancement in the outcomes after septoplasty.

About 34 patients complained of ear fullness in the preoperative assessment, which agrees with the findings of Nanda et al. 4 and Abdel-Naby Awad et al. 16 Similar results were obtained by Moorthy et al. 17 who found that DNS affects preoperatively a significant result had negative pressure of the middle ear found as a type-C tympanogram curve on tympanometry.

This suggests that DNS leads to blockage of the ET, which impacts the function of the middle ear. It had been reported in the literature that septal deviation could lead to ET dysfunction, leading to negative middle ear pressure. 8

In the current study, patients reported that ear fullness was markedly improved after septoplasty, which agrees with the result reported in the study by Nanda et al. 4

In addition, the present study shows the middle ear function evaluated by tympanometry, which renders specific graphs for ear pathologies. A type-C tympanogram curve reflects ET dysfunction, which is a frequent set of malfunctions of middle ear sub-clinically. Even a negative middle ear tube pressure on tympanometry, especially when the peak is below -100 daPa, can indicate ET dysfunction. The same finding was reported by El-bary et al., 18 who reported that there is a significant improvement in type of tympanometry from C to A, and the middle ear pressures showed significant improvement after surgery in both ears.

In the current study, the Arabic version of the ETDQ-7 questionnaire was validated, as shown in Fig. 1 .

Alkholaiwi et al., 19 in their study, concluded that the Arabic version of the ETDQ-7 scale is a valid instrument for evaluating the severity of the ET dysfunction, and it is used as an important tool for diagnosis, follow-up, and treatment management.

In addition, Rathaur and Verma 20 concluded that surgery, such as septoplasty, for nasal obstruction has a favorable impact on the pressure of the middle ear and ETF.

However, a study performed by Eyigör et al. 21 showed that the success of septoplasty did not have significant impact on the pressure or ventilation of the middle ear.

The current study had several limitations, such as the fact that it was conducted at a single center and the relatively small sample size.

## Conclusion

The current study detected that DNS has a negative effect on middle ear ventilation ETF. After septoplasty, there were statistically significant findings, such as reduction in the ETFQ-7 scores, number of ears with ET dysfunctions, and mean tympanometry peak pressures measurements. In addition, there was marked improvement regarding the symptom of ear fullness, as well as tympanometry curve changes, mainly from type C to type A.

Therefore, the septoplasty technique results in improvement of middle ear ETF.
